# miR-663 sustains NSCLC by inhibiting mitochondrial outer membrane permeabilization (MOMP) through PUMA/BBC3 and BTG2

**DOI:** 10.1038/s41419-017-0080-x

**Published:** 2018-01-19

**Authors:** Micol E. Fiori, Lidia Villanova, Chiara Barbini, Maria Laura De Angelis, Ruggero De Maria

**Affiliations:** 1 Institute of General Pathology, Catholic University of the Sacred Heart and Gemelli Polyclinic, Rome, Italy; 2 Department of Oncology and Molecular Medicine, Istituto Superiore di Sanità, Rome, Italy

## Abstract

Treatment of lung cancer is an unmet need as it accounts for the majority of cancer deaths worldwide. The development of new therapies urges the identification of potential targets. MicroRNAs’ expression is often deregulated in cancer and their modulation has been proposed as a successful strategy to interfere with tumor cell growth and spread. We recently reported on an unbiased high-content approach to identify miRNAs regulating cell proliferation and tumorigenesis in non-small cell lung cancer (NSCLC). Here we studied the oncogenic role of miR-663 in NSCLC biology and analyzed the therapeutic potential of miR-663 targeting. We found that miR-663 regulates apoptosis by controlling mitochondrial outer membrane permeabilization (MOMP) through the expression of two novel direct targets PUMA/BBC3 and BTG2. Specifically, upon miR-663 knockdown the BH3-only protein PUMA/BBC3 directly activates mitochondrial depolarization and cell death, while BTG2 accumulation further enhances this effect by triggering p53 mitochondrial localization. Moreover, we show that miR-663 depletion is sufficient to elicit cell death in NSCLC cells and to impair tumor growth in vivo.

## Introduction

Lung cancer is a disease with enormous implications on public health as it is nowadays the foremost cause of cancer-related death^1^. Despite advances in diagnostics, the improvement of surgical procedures and the development of new biologic drugs, the overall survival rate for lung cancer remains frustratingly poor^2,3^.

Lung cancer derives from aberrant cells of the respiratory epithelium. It is a heterogeneous disease that can be divided into two subtypes: small cell lung cancer (SCLC), a highly malignant tumor developed from neuroendocrine cells and non-small cell lung cancer (NSCLC). NSCLC accounts for the 85% of patients and is further divided into three major histotypes: adenocarcinoma, squamous cell carcinoma, and large cell carcinoma. To date the vast majority of patients are diagnosed at advanced stages and available therapies are often only temporarily effective. The study of the molecular basis of lung cancer, aimed at the identification of novel therapeutic targets is a major need to fight this devastating disease^4^.

MicroRNAs (miRs) are a class of highly conserved non-protein-coding RNAs, involved in the regulation of gene expression^5^. In mammals, single-stranded miRs bind to partially complementary target sequences on specific mRNAs (usually in the 3′ untranslated region -3′ UTR) thus inhibiting translation and/or promoting mRNA degradation^6^. In the last decades, the deregulation of miR function has been linked to cancer occurrence, progression and metastasis^7,8^. In particular, it has been shown that miRs can act as oncogenes and/or oncosuppressors in a tissue-specific manner, envisaging the possibility to develop new anti-cancer strategies based on miRs’ expression modulation. The ability of miRs to orchestrate a complex network of interactions by concurrent targeting of several mRNAs endows these molecules with a great regulatory potential, allowing a kind of resetting of the cellular fate by interfering with a single miR^9^.

Programmed cell death or apoptosis is a pivotal mechanism in the development of multicellular organisms and tissue homeostasis maintenance. Inappropriate apoptosis leads to several human pathologies including many types of cancer. Tumor spread entails the escape from apoptotic response and regulators of apoptosis are often mutated in cancer^10,11^. Abundant literature has highlighted that miRs regulate apoptosis at different levels, envisaging the possibility to control cell death through miR modulation.

We have recently investigated the role of all known miRNAs in NSCLC through the neutralization of their function, taking advantage of a locked nucleic acid (LNA)-based anti-miRNA library (miRCURY LNA microRNA inhibitor library, Exiqon, Vedbaek, Denmark)^12^. We identified a number of miRs whose knock-down impairs NIH-H460 lung cancer cell survival and/or proliferation and classified them as bona fide oncogenes. Among the top 20 hits, we focused on miR-663 showing that it is a key regulator of the apoptotic cascade through its direct targets PUMA/BBC3 (p53 up-regulated modulator of apoptosis/Bcl-2 binding component 3) and BTG2 (B-cell translocation gene 2). In NSCLC miR-663 allows cancer cells to escape apoptosis, thus promoting tumor onset and propagation. We propose miR-663 as novel therapeutic target as its neutralization leads to cell death in vitro and reduces tumor growth in vivo.

## Results

### miR-663 inhibition impairs NSCLC cell proliferation and tumor growth in vivo

**Fig. 1:**
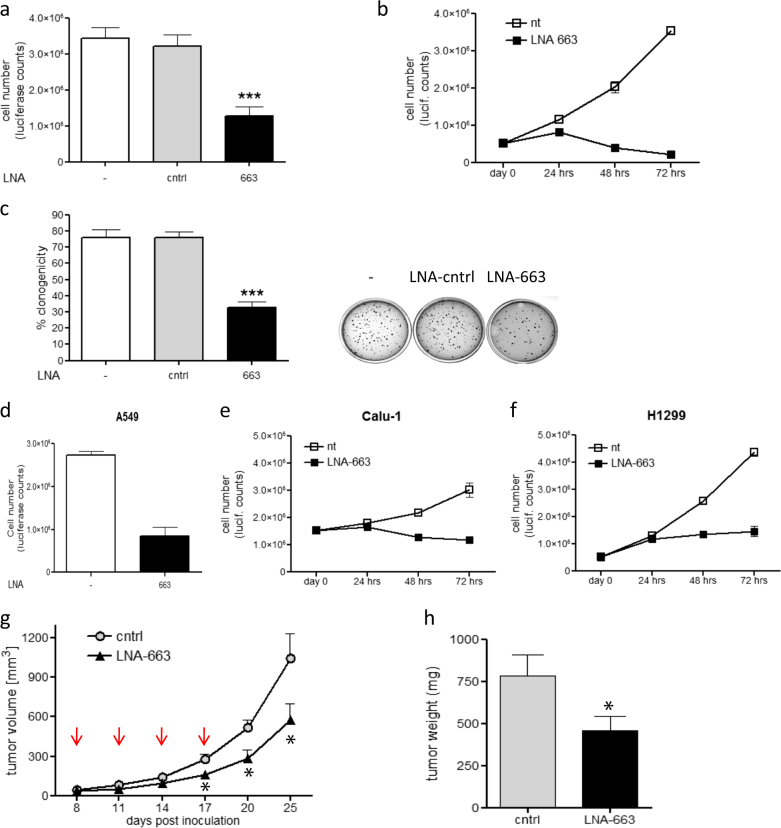
miR-663 inhibition impairs cell viability and tumorigenic potential of NSCLC cells **a** Cell viability of NIH-H460 cells measured 72 h post transfection with 25 nM cntrl LNA or LNA-663. **b** Growth curve of NIH-H460 cells untreated (nt) and transfected with LNA-663 at 25 nM; as in panel (**a**), cell number was assessed by Cell Titer Glo assay at the indicated time points after transfection. **c** Clonogenic capacity of NIH-H460 cells after anti-miR-663 treatment; the graph shows the percentage of plated cells that gave rise to colonies. **d** Cell viability of A549 cells measured 72 h post transfection with LNA-663 (100 nM). **e**, **f** Growth curve of Calu-1 and H1299 cells untreated (nt) and transfected with LNA-663 at 25 nM. **g** 2*10^5^ NIH-H460 cells were injected in the flank of CD1/nude mice. After tumors had reached a palpable volume, LNA-663 was locally administered (15 mg/kg) at the indicated time points (red arrows). Shown is the tumor growth of LNA-treated xenografts versus PBS vehicle, as defined by mass volume—mean ± s.e.m., *n* = 8. **h** Tumors were explanted and weighted at the end of the experiment. Shown is the mean tumor weight for each group. Mean ± s.e.m., *n* = 8 *** *p* < 0.001, *p < 0.05

As a result of anti-miR-LNA library screening in NIH-H460 cells, miR-663 listed among the top 20 hits endowed with oncogenic features. We first performed some in vitro experiments to confirm the screening results. Neutralization of miR-663 by LNA transfection was able to impair NIH-H460 cells viability (Fig. 1a, b) and anchorage-independent growth (Fig. 1c), inferring a pro-tumoral role of miR-663. We tested whether miR-663 function was necessary for other NSCLC cells by knock-down experiments in A549, Calu-1, and H1299 cells (Fig. 1e, f and Fig. S1). The four assayed cell lines represent different histological subtypes of NSCLC, distinct tissues of origin and diverse mutational status (Table S1). As shown in Fig. 1 (panels d–f), LNA-663 treatment significantly reduced cell growth in all the systems tested, suggesting that this miRs commonly behaves as an oncogene in NSCLC. Further, we tested the effect of miR-663 depletion in patient-derived cells grown as tumorspheres, confirming the pro-tumoral role of miR-663 in this system (Fig. S2a and data not shown). We next investigated if interfering with miR-663 function could represent an effective strategy to hamper tumor growth in vivo. To test this hypothesis CD1 nude mice were subcutaneously injected with NIH-H460 cells. When tumors became palpable (~40 mm^3^), LNA-663 was locally administered every 3 days, in four doses of 15 mg/kg. Growth curve of LNA-663-treated versus control tumors showed a significant reduction in mass expansion that lasted after treatment completion, as shown also by tumor weight measured ex vivo (Fig. 1g, h and Fig. S1e).

### miR-663 controls apoptosis in NSCLC cells

**Fig. 2:**
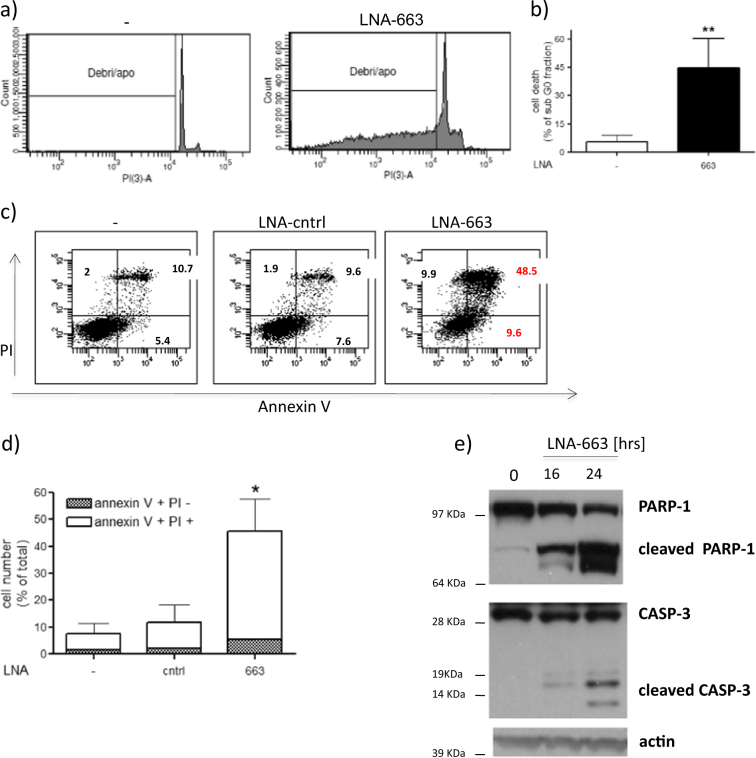
miR-663 depletion induces apoptosis The percentage of apoptotic NIH-H460 cells upon transfection with LNA-663 at 25 nM was analyzed by FACS and is shown as sub-G_0_ fraction. **a** one representative experiment, **b** mean of four independent experiments. Mean ± s.d. Annexin V staining showing apoptosis induction 72 h after LNA-663 transfection; **c** one representative experiment, **d** mean of two independent experiments. Mean ± s.d., **e** PARP-1 and CASPASE 3 cleavages at the indicated time points after LNA-663 transfection is shown by immunoblot.**p < 0.01, *p < 0.05

Sustained by the above data, we deepened our analysis to better understand the role of miR-663 in NSCLC. FACS analysis of miR-663 knockdown cells showed an increase of the sub G_0_ fraction, suggestive of apoptosis (Fig. 2a, b). Annexin V staining confirmed that miR-663 inhibition triggers programmed cell death (Fig. 2c, d). Moreover, the appearance of cleaved PARP-1 and cleaved Caspase 3 already 16–24 h post LNA-663 transfection demonstrated that the kinetics of apoptotic cascade induction is extremely rapid (Fig. 2e), suggesting a direct effect on cell death effectors. Apoptosis induction upon miR-663 deprivation was observed also in other lung cancer cell lines (Fig. S2). Altogether, these data indicate that the oncogenic role of miR-663 in NSCLC cells implicates the control of apoptosis.

### PUMA is a direct target of miR-663

In order to identify miR-663 targets that mediate apoptosis induction, in silico analyses were conducted taking advantage of the algorithms TargetScan, RNA22 and miRanda. In particular, we focused on positive regulators of apoptosis and selected PUMA/BBC3 among predicted targets. Identified in 2001, this gene was characterized as activator of both p53-dependent and p53-independent apoptosis^13–17^. It belongs to the BH3-only subclass that regulates canonical mitochondrial apoptosis: upon internal or external stimuli, PUMA binds to inhibitory members of Bcl-2 protein family displacing Bak and Bax and thus triggering mitochondrial outer membrane permeabilization (MOMP) and caspase activation^18,19^. Interestingly, the genomic region 19q13.3 entailing the *PUMA/BBC3* gene is often deleted in human cancer^13,18,20^.

**Fig. 3:**
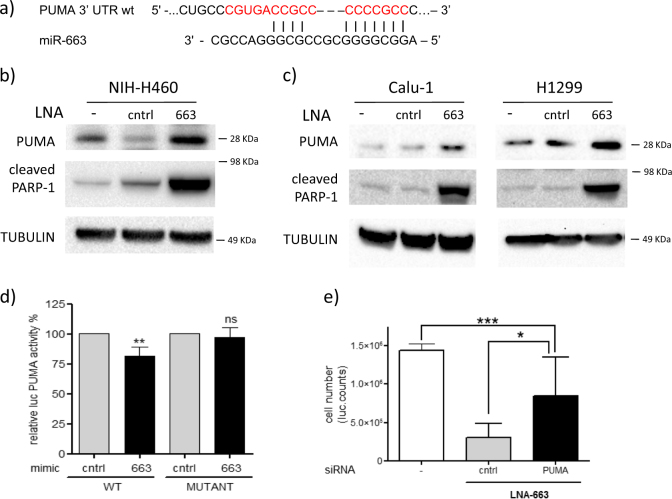
PUMA is a direct target of miR-663 **a** Predicted PUMA 3′UTR binding site for miR-663. The alignment of the seed region of miR-663 with PUMA 3′ UTR is shown. The sites of target mutagenesis are indicated in red. **b**, **c** Immunoblot showing inverse correlation of PUMA and miR-663 expression paralleled by PARP-1 cleavage in NIH-H460 **b** Calu-1 and H1299 cells **c**. **d** Luciferase activity in HeLa cells transfected with PUMA UTR wt or mut in combination with a control or miR-663 mimic. The normalized luciferase activity is indicated—mean** ± **s.e.m., **p = 0.008. **e** Downmodulation of PUMA by RNA interference can rescue LNA-663 apoptotic effect in NIH-H460 cells as shown by Cell Titer Glo assay. Mean ± s.d., ***p < 0.001, *p < 0.05

In Fig. 3a is shown the putative miR-663-binding site on PUMA 3′UTR. The inverse correlation of expression of miR-663 and PUMA was demonstrated by knock down experiments: a marked increase in protein level was found when cells were treated with LNA-663 (Fig. 3b). To evaluate the possible contribution of transcriptional activation by p53 to PUMA accumulation, mRNA levels were monitored by qPCR. Surprisingly, despite overall p53 stabilization observed by western blot, PUMA mRNA levels resulted unaffected by miR-663 modulation, corroborating the hypothesis of a miR-dependent post-transcriptional regulation (Fig. S3a-b). Further, PUMA upregulation upon miR-663 depletion was observed also in p53-NULL cells Calu-1 and H1299 together with PARP-1 cleavage, indicative of apoptosis (Fig. 3c). To verify the direct interaction between miR-663 and PUMA mRNA, the 3′UTR was cloned downstream of the renilla luciferase gene in the psiCHECK-2 vector (Promega) and co-transfected with a miR-663 mimic. Furthermore, to validate target specificity, PUMA 3′UTR mutant derivative was generated where the putative miR-663 binding site was deleted (base pairing with miR-663 seed sequence was abolished by deleting the nucleotides marked in red—Fig. 3a). Reduction in luciferase activity upon miR-663 overexpression was observed in the presence of wild type PUMA 3′UTR, while the mutated construct was unaffected by miR-663 levels, indicating that PUMA is indeed a bona fide target of miR-663 (Fig. 3d). PUMA knock-down by siRNA was able to relieve the LNA-663-induced phenotype on cell viability, further highlighting the pivotal role of this target in apoptosis induction (Fig. 3e and Fig. S3c). However, the partial rescue obtained by this approach suggests a cooperative action of PUMA with other unidentified miR-663 targets.

### BTG2 is a novel direct target of miR-663

To better characterize the network of molecular interactions orchestrated by miR-663 and possibly identify novel direct targets, we performed transcriptome analyses by microarrays in NIH-H460 cells treated with LNA-663 (Whole Transcript Expression Arrays, HuGene 1.0 St, Affymetrix).

**Fig. 4:**
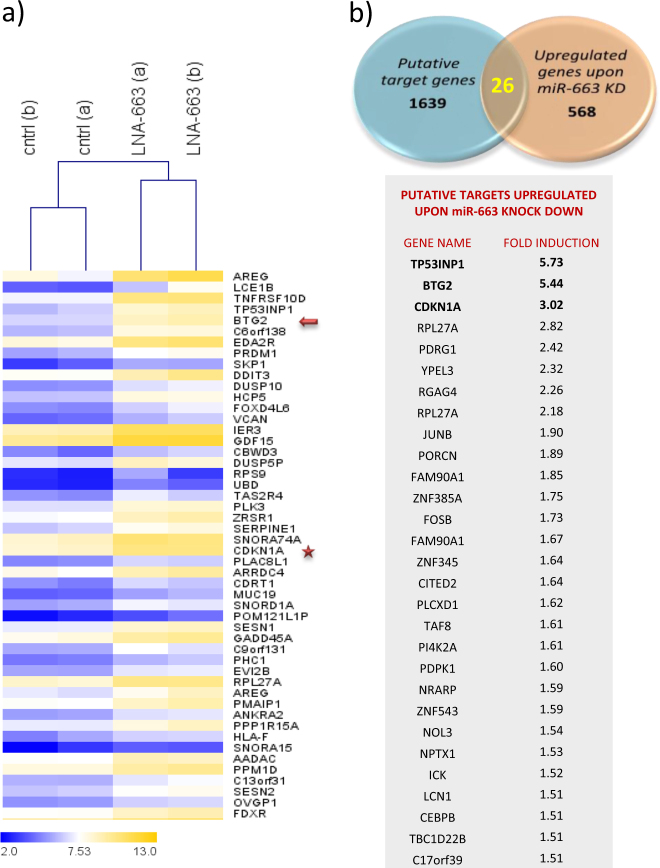
Identification of novel miR-663 target genes **a** Unsupervised hierarchical clustering of genes that are significantly upmodulated upon miR-663 depletion (≥1.5 fold increase); genes in the heatmap are shown starting from the most upregulated to the most downmodulated one. The top 50 genes are shown. The arrow indicates BTG2 and the star CDKN1A/p21. **b** Venn diagram showing the integrated analysis of putative miR-663 target genes and top 568 upregulated genes ( > 1.5 fold) upon miR-663 knock down. Twenty-six identified putative target genes of miR-663 are listed in the table together with relative fold increase as measured by gene arrays

We used an integrative approach combining microRNA target predictions by TargetScan (release 5.2) and the list of 568 genes that resulted upregulated ≥1.5 fold by gene arrays upon miR-663 depletion (Fig. 4a, b and Tables S2, S3). As a positive control of our system, we employed the miR-663 known target p21 (CDKN1A) that resulted upregulated upon miR knockdown (3.02 mean fold induction, *p* < 0.01) and ranked at third position in overlaid datasets (Fig. 4a, b and Tables S2,3). We first confirmed p21 protein accumulation after miR-663 knock down (Fig. S4a). Moreover, we found that p21 mediates the effect of LNA-663 on NSCLC cells, as impairment of p21 upregulation by siRNAs was able to partially rescue cell viability/proliferation of miR-663-depleted cells (Fig. S4b). In nasopharyngeal carcinoma, it was reported that induction of p21 upon miR-663 knockdown leads to cell cycle arrest in G1 phase, while no apoptosis was described^21^. We therefore assumed that the rescue observed by combining LNA-663 with siRNAs against p21 is due to miR-663 regulation of G1/S phase transition and thus to an increased cell proliferation rather than to the reduction of cell death. Since in NSCLC miR-663 inhibition leads to a quick and massive induction of apoptosis, we reasoned that p21 plays a marginal role and focused our efforts on novel hypothetical targets controlling programmed cell death.

**Fig. 5:**
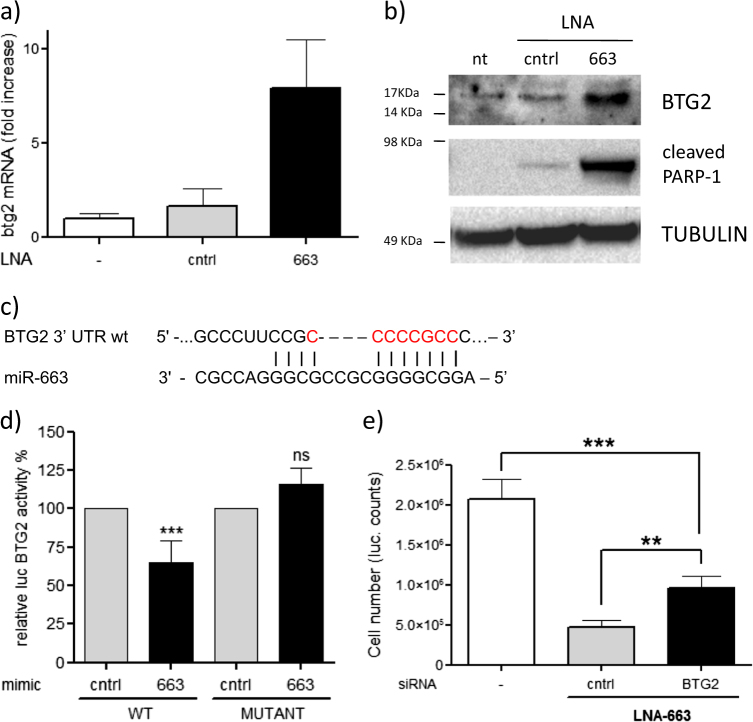
BTG2 is a direct target of miR-663 **a** q-RT-PCR showing inverse correlation of BTG2 mRNA and miR-663 expression. GAPDH was used for PCR normalization—mean** ± **s.d.; **b** Western blotting showing the upmodulation of BTG2 protein upon miR-663 neutralization, paralleled by PARP-1 cleavage. TUBULIN was used as protein loading control. **c** Predicted BTG2 3′ UTR binding site for miR-663. The alignment of the seed region of miR-663 with BTG2 3′ UTR is shown. The sites of target mutagenesis are indicated in red. **d** Luciferase activity in HeLa cells transfected with BTG2 UTR wt or mut in combination with a control or miR-663 mimic. The normalized luciferase activity is indicated—mean** ± **s.e.m.***p = 0.0004. **e** Downmodulation of BTG2 by RNA interference can rescue LNA-663 apoptotic effect in NIH-H460 cells as shown by Cell Titer Glo assay—mean ± s.d.***p < 0.001, *p < 0.05

The first putative target upregulated upon miR-663 depletion was TP53INP1 (tumor protein p53 inducible nuclear protein 1), an antiproliferative and proapoptotic protein induced by p53 that acts as a mediator of cell response to stress^22–24^. TP53INP1 protein levels resulted unaffected by miR-663 knock down concluding that it is not directly modulated by this microRNA (Fig. S4c,d). The second candidate in our combined list was the putative target BTG2, which listed among top five upregulated genes (5.44 mean fold induction, *p* < 0.01) suggesting a possible direct correlation with miR-663 levels (Fig. 4a, b). Consistently, knockdown of miR-663 by LNA increased BTG2 expression at both mRNA and protein levels in NIH-H460 cells (Fig. 5a, b). The role of BTG2 has been extensively studied. BTG2 acts through multiple mechanisms regulating transcription, differentiation, and development^25–27^. Moreover, it is an oncosuppressor gene in cancer cells, controlling cell cycle progression, DNA damage repair, and apoptosis. Noteworthy, BTG2 expression was found downregulated in many human cancers^28–32^.

To verify whether miR-663 directly binds to BTG2 3′UTR, the region flanking the miR putative binding site was cloned in psiCHECK-2 vector and luciferase assay was performed. A mutant construct lacking the eight nucleotides recognized by the miR seed sequence was used as a control (Fig. 5c). Co-transfection of wt UTR together with a miR-663 mimic in HeLa cells caused a consistent decrease of luciferase activity, that was abolished when using the mutant construct (Fig. 5d), demonstrating that miR-663 indeed targets BTG2.

### BTG2 plays an essential role in miR-663-mediated apoptosis control

**Fig. 6:**
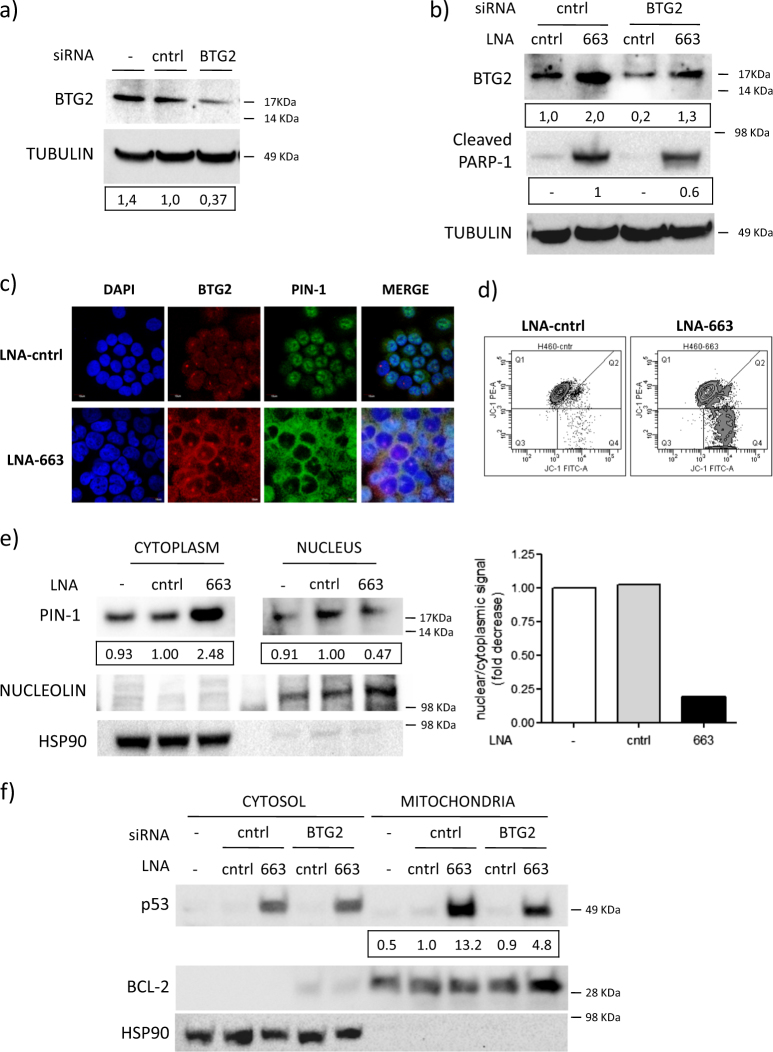
miR-663 controls PIN-1-mediated apoptosis and p53 mitochondrial function **a** Immunoblot showing BTG2 downmodulation 72 h after siRNA transfection. TUBULIN was used as protein loading control. Normalized BTG2 levels are expressed as fold decrease relative to cntrl treated cells. **b** Western blotting showing BTG2 modulation in NIH-H460 after the indicated treatments. Double transfection of anti-BTG2 siRNA together with LNA-663 restores BTG2 levels of control cells, counteracting LNA-mediated induction. PARP-1 cleavage is reduced to 60%. TUBULIN was used as protein loading control. Numbers indicate BTG2 levels fold change relative to cntrl treated cells, and cleaved PARP-1 levels fold decrease relative to LNA-663/cntrl siRNA-treated cells. The values were normalized to TUBULIN signal, as measured by densitometric analyses. **c** Subcellular BTG2 and PIN-1 localization in NIH-H460 cells treated with control LNA or LNA-663 was assessed by immunofluorescence staining. **d** Mitochondrial membrane potential of NIH-H460 cells upon miR-663 downmodulation was measured by JC-1 staining. An increase of the ratio between green and red signals is indicative of membrane depolarization typical of apoptotic cells. **e** Western blotting showing PIN-1 modulation in NIH-H460 subcellular fractions 24 h after LNA-663 treatment. Cytoplasmic accumulation and nuclear decrease of PIN-1 are expressed as fold changes relative to cntrl-treated cells. The histogram shows the ratio between cytoplasmic and nuclear signal. **f** Immunoblot of p53 in NIH-H460 subcellular fractions. Hsp90 and Bcl-2 were used as cytoplasmic and mitochondrial markers, respectively. Mitochondrial p53 modulation is expressed as fold increase relative to cntrl treated cells and normalized to Bcl-2 signal

To evaluate the role of BTG2 in miR-663-mediated control of apoptosis, we neutralized LNA-663-induced BTG2 overexpression by siRNAs (Fig. 6a, b). NIH-H460 cells treated with both LNA-663 and BTG2 siRNAs showed a milder reduction of cell viability if compared with LNA-663-treated cells, suggesting that this target actively determines the apoptotic phenotype (Fig. 5e). It has been previously shown that BTG2 overexpression can lead to programmed cell death through direct binding to PIN-1 (protein-interacting NIMA): upon BTG2 binding, PIN-1 translocates from nucleus to cytoplasm, where it leads to mitochondrial membrane depolarization^33,34^. PIN-1 interacts with and regulates the activity of several proteins by inducing cis-trans isomerization. Noteworthy, PIN-1 is considered a master regulator of mitochondrial apoptosis^35^. One of the mechanisms of apoptosis induction by PIN-1 involves a non-canonical role of p53. In particular, it has been shown that PIN-1 induces a conformational change of cytoplasmic p53, thus reducing its affinity for MDM2 and leading to cytoplasmic accumulation of mono-ubiquitinated p53. Under these conditions, p53 translocates to the mitochondrial membrane, where it behaves as a BH3-only protein interacting with Bcl2 family members and inducing MOMP, with a kinetics that is faster than p53 transcriptional program activation^34–39^. Cytoplasmic PIN-1 is therefore regulating transcription-independent p53 induction of apoptosis.

To determine whether miR-663 inhibition was sufficient to affect PIN-1 localization, we conducted immunofluorescence analyses, which showed that downmodulation of miR-663 induces BTG2 overexpression and cytoplasmic diffusion that is accompanied by PIN-1 nuclear exit, as confirmed also by western blot (Fig. 6c, e and Fig. S5). These events eventually lead to mitochondrial membrane depolarization characteristic of apoptosis, as shown by monitoring mitochondrial membrane potential with JC-1 staining (Fig. 6d). By western blot analyses of intracellular fractions we observed overall p53 stabilization and concomitant mitochondrial localization upon miR-663 depletion (Fig. S2a and Fig. 6f). To establish whether this process is indeed dependent on BTG2, we performed knockdown experiments. siRNAs significantly reduced BTG2 expression while double treatment with LNA-663 and siRNAs restored BTG2 protein levels of control cells, counteracting LNA-663-mediated BTG2 induction (Fig. 6a, b). Under these conditions, p53 accumulation on mitochondria is impaired and apoptosis induction decreased (Fig. 6b–e). Further we explored BTG2 role in the absence of p53 to establish whether other BTG2-dependent mechanisms (involving or not PIN-1) are relevant for apoptosis induction^35^. We verified that in p53-NULL cells BTG2 is upregulated upon miR-663 downmodulation (Fig. S6a–d). However, by RNA interference we were not able to rescue the effect on cell viability of LNA-663 treatment (Fig. S6e–h), suggesting that the role of BTG2 is not relevant in the absence of functional p53.

In conclusion, these data reveal that miR-663 exerts a pivotal role in apoptosis regulation of NSCLC cells, controlling convergent mechanisms of MOMP through the identified target genes PUMA and BTG2.

## Materials and methods

### Cell culture

Human NSCLC A549 and Calu-1 (American Type Culture Collection—ATCC, Manassas, VA, USA) were grown in DMEM, whereas HeLa, NIHH460, and H1299 (ATCC) in RPMI 1640 (Lonza, Basel, Switzerland). All cell media were supplemented with 10% heat-inactivated fetal bovine serum (PAA, Pasching, Austria). Patient-derived NSCLC cells were isolated from tumor specimens and grown in suspension as tumorspheres in a serum-free medium supplemented with 20 ng/ml EGF and 10 ng/ml bFGF (Peprotech), as previously described^40^.

### Transfection experiments

Transfection experiments with oligonucleotides (siRNAs, miR-mimics, and LNAs) were performed using HiPerFect transfection reagent (Qiagen), following manufacturer’s instructions. LiPofectamine 2000 (Invitrogen) was used for transfections with DNA constructs. All siRNAs were used at the final concentration of 25 or 50 nM. PUMA/BBC3 siRNAs were purchased from Qiagen (FlexiTube siRNAs #2 and #3, SI02655520 and SI02822820, respectively), whereas BTG2 siRNA from Dharmacon (ON-TARGET plus, Set of 4 Upgrade LU-012308-00, siRNA #5). The miR-mimics (Qiagen) were used at the final concentration of 30 nM and the LNAs (Exiqon) at the final concentration of 25 or 50 nM.

### Anchorage-independent growth assay

Cells were seeded in 24-well plates and transfected with indicated LNAs at the final concentration of 25 nM. Twenty-four hours post-transfection cells were trypsinized and counted with trypan blue exclusion. A total of 500 healthy cells were resuspended in 1.5 ml semisolid medium consisting of RPMI 1640 supplemented with 10% FBS and 0.3% Agar Noble (Difco, Kansas City, Missouri) and plated on solidified 1.5 ml RPMI 1640 supplemented with 10% FBS and 0.6% Agar Noble. Colonies formation was assessed after 2 weeks upon crystal violet staining (Fluka, St. Gallen, Switzerland) and manual counting at the microscope.

### Cell proliferation and apoptosis detection

For proliferation assay, cells were seeded at the same density (2 × 10^3^ cells/well) in 96-well plate and transfection was performed using HiPerFect reagent (Qiagen) and 50 nM LNAs (Exiqon). At the indicated time points, three wells for each condition were analyzed. Growth curves were generated by evaluating cell number with CellTiter-Glo Luminescent Cell Viability Assay (Promega), following manufacturer’s instructions.

Apoptosis was measured with the Apoptosis Detection Kit (MBL International, Woburn, MA). Briefly, 4 × 10^4^ cells were seeded in 24-well plate and transfected with control LNA and LNA-663; at the indicated time points cells were harvested, counted, and stained with Annexin V-FITC and PI (5 µg/ml) and analyzed using a BD FACS Canto Cytometer (Becton Dickinson, San Jose, CA). In addition, the percentage of apoptotic cells was assessed also by detecting the sub-G_0_ fraction upon incubation with Nicoletti buffer (0.1% sodium citrate, pH 7.4, 0.1% NP40, 9.65 mM NaCl, 200 mg/ml RNAse A, 50 mg/ml PI) and FACS analysis.

### Immunoblotting

Whole cell protein extracts (lysis buffer: 30 mM Tris–HCl pH 7.5, 150 mM NaCl, 2 mM KCl, 2 mM EDTA, 5% Glycerol, 0.1% Triton X-100, and 1× Protease Inhibitor Cocktail—Sigma-Aldrich, St. Louis, MO) were quantified by BCA assay (Pierce, Rockford, IL), separated onto NuPAGE 4–12% polyacrylamide gels (Invitrogen, Carlsbad, CA) and blotted on nitrocellulose membranes (Amersham, England). Membranes were hybridized with polyclonal antibodies anti-PARP-1 (#9542 Cell Signaling Technology, MA), anti-Caspase-3 (#9665 Cell Signaling Technology), anti-PUMA/BBC3 (#4976 Cell Signaling Technology, MA), anti-HSP90 (#4877 Cell Signaling Technology, MA), anti-Lamin-A/C (#sc-6215 Santa Cruz Biotechnology), anti-p21 (#sc-756, Santa Cruz Biotechnology), anti-TP53INP1(#NBP1-76638 Novus-Biologicals) or monoclonal antibodies anti-Bcl-2 (#sc-509 Santa Cruz Biotechnology), anti-BTG2 (#SAB1404558 Sigma-Aldrich), and anti-p53 (#sc-126 Santa Cruz Biotechnology). Monoclonal antibody either anti-Actin (#CP01, Calbiochem) or anti-α-Tubulin (#T5168 Sigma-Aldrich) was used as a loading control. Bands were visualized and quantified with FluorChem E System (Protein Simple, CA) or with ChemiDoc XRS+ and ImageLab^TM^ software (Bio-Rad Laboratories, Inc, Hercules, CA).

### DNA constructs

*PUMA/BBC3* 3′UTR was PCR amplified from human genomic DNA by using the following primers: *PUMA/BBC3*-3′UTR-for 5′-GATCTCGAGCACTGACGGAGATCGCCA-3′, *PUMA/BBC3*-3′UTR-rev 5′-ATAGCGGCCGCTCTACAGCAGCGATATACA-3′. The PCR fragment was cloned downstream of the Renilla luciferase gene in the psiCHECK-2 vector (Promega) by digestion with XhoI and NotI. The mutant derivatives, lacking the putative miR-663 binding sites, were generated from these constructs by inverse PCR with the following primers: *PUMA/BBC3*-Δ-for 5′-TTCCCATCAATCCCATTGCATAG-3′ and *PUMA/BBC3*-Δ-rev5′-TTCGGTCACGGGCAGAGCACAGG-3′.

Short oligonucleotides (~70 nucleotides in length) spanning the putative miR-663 binding sites within *BTG2* 3′UTR were designed as follows: *BTG2*-3′UTR-for5′-TCGAGGAACTACGTGATGGCAGTCTCCAGCTAGGCCCTTCCGCCCCCGCCCTGGGCGCCGCCGTGCTCATGCTGCCGTGGC-3′, *BTG2*-3′UTR-rev5′-GGCCGCCACGGCAGCATGAGCACGGCGGCGCCCAGGGCACGTAGTTCC-3′. For the mutant derivatives, oligonucleotides lacking the putative miR-663-binding sites were used: *BTG2*-Δ-for5′-AGTCTCCAGCTAGGCCCTTCC-3′ and *BTG2*-Δ-rev 5′-GGCCGCCACGGCAGCATGA GCACTGCCATCACGTAGTTCC-3′. Briefly, psiCHECK-2 vector was digested with XhoI and NotI and its ends dephosphorylated with CIP (M0290S, New England BioLabs) in order to prevent re-ligation of the linearized vector. Each pair of oligos was annealed upon phosphorylation with T4 PNK (Poly Nucleotide Kinase—New England BioLabs) and cloned downstream of the Renilla luciferase gene.

### Luciferase reporter assay

In luciferase experiments, HeLa cells were seeded in 96-well plate (4 × 10^4^ cells/well) and transfected with 40 ng of Firefly/Renilla luciferase vectors (empty psiCHECK-2, psiCHECK-2-PUMA/BBC3 3′UTR wt or mutant, psiCHECK-2-BTG2 3′UTR wt or mutant) together with 30 nM miR mimics (Qiagen, Germany). 0.2 µl/well of Lipofectamine 2000 (Invitrogen) were used for transfection. The activity of both Firefly and Renilla luciferases was measured 72 h post-transfection using the Dual Luciferase Assay kit (Promega) and the luminescence plate reader Victor-X3 (Perkin Elmer, MA). Transfection efficiency was normalized by calculating the ratio firefly/renilla. The experiment was performed three times in quadruplicate.

### q-RT-PCR analysis

For mRNA analysis, NIH-H460 cells were treated with 10 nM LNA-663 for 92 h and total RNA was purified with TRIZOL Reagent (Invitrogen) and reverse transcribed with random primers (N6, Roche, Switzerland) and M-MLV RT (Invitrogen) after DNase-I treatment (RQ1 DNase, Promega). Real-time PCR was performed with SensiMix SYBR Hi-ROX (Bioline) for both SYBR Green- and Taqman probes-based assays. *BTG2* cDNA was amplified using the following primers: BTG2-for 5′-GCGTGAGCGAGCAGAGGCTT-3′ and BTG2-rev 5′-GGCTGGCCACCCTGCTGATG-3′. *PUMA/BBC3* cDNA was amplified using a gene-specific Taqman probe assay (Applied Biosystems, CA). For miR-663 analysis, we proceeded as in Pan et al.^41^ Briefly, total RNA was purified with TRIZOL Reagent and reverse transcribed with the following miR-663-specific stem-loop primer: 5′- GTCGTATCCAGTGCGTGTCGTGGAGTCGGCAATTGCACTGGATACGACGCGGTCC-3′. MiR-663 cDNA was amplified using the following primers: miR-663-for 5′- GTGCGTGTCGTGGAGTCG-3′ and miR-663-rev 5′-TTTAGGCGGGGCG-3′.

Samples were run on an ABI PRISM 7900HT Sequence Detection System (Applied Biosystems, CA) according to standard procedures. Human GAPDH and U6-snRNA were used as endogenous control.

### Immunofluorescence

2 × 10^4^ NIH-H460 cells were seeded in each chamber polystyrene vessel (BD Falcon) and transfected with LNA-663 and relative control at the final concentration of 50 nM. Seventy-two hours post-transfection the cell medium was removed and cells were washed once with PBS and fixed in 2% paraformaldehyde for 10 min at RT. Cells were permeabilized by incubation with PBS + 0.1% Triton X-100 for 15 min at RT, and blocking was performed using PBS + 3% bovine serum albumin (BSA) for 1 h at RT. Anti-BTG2 (#SAB1404558 Sigma-Aldrich) and anti-PIN-1 (sc-15340 Santa Cruz Biotechnology, CA) antibodies were used 1:100 in PBS + 3% BSA. Goat anti-Mouse IgG (H + L) Alexa Fluor® 647 conjugate and Goat anti-Rabbit IgG (H + L) Alexa Fluor® 488 conjugate secondary antibodies (Life technologies) were diluted 1:1000 together with 1:20,000 DAPI (4′,6-Diamidino-2-Phenylindole, Dihydrochloride, Life technologies) in PBS and incubated with cells for 1 h at RT. Coverslips were then mounted on glass slides with ProLong Gold Antifade Mountant (Life technologies) and images were acquired on a confocal microscope (Olympus, Japan). Nuclear and cytoplasmic signals were quantified with ImageJ.

### Measurement of mitochondrial membrane potential

Changes in the mitochondrial membrane potential (Δ*Ψ*_mt_) were analyzed by flow cytometry using the Δ*Ψ*_mt_-sensitive dye 5,5′,6,6′-tetrachloro-1,1′,3,3′-tetraethylbenzimidazolylcarbocyanine iodide (JC-1, Molecular probes, Life technologies). In highly polarized mitochondria JC-1 accumulates as red fluorescent aggregates. Apoptosis results in a depolarization of the mitochondrial membrane that disrupts JC-1 aggregates and can be visualized as a decrease in the red fluorescence and a shift towards a green fluorescence, yielded by the JC-1 monomers. NIH-H460 cells were seeded in six-well plates and transfected with LNA-663 and control LNA at the final concentration of 50 nM. Seventy-two hours post-transfection, cells were trypsinized and counted; 1.5*10^5^ cells were harvested, washed once in PBS and incubated for 15 min at 37 °C upon addition of 5 µg/ml JC-1. After washing twice in PBS, two fluorescence parameters, fluorescence channel 1 (FL1) for green fluorescence and fluorescence channel 2 (FL2) for red fluorescence, were measured by fluorescence-activated cell sorting (FACS).

### Subcellular fractionation

NIH-H460 cells were seeded in six-well plates and treated with 25 nM LNA-663 or control LNA for 28 h. 1 × 10^6^ cells were harvested for subcellular fractionation. For isolation of the mitochondrial fraction we used the Qproteome Cell Compartment Kit (Qiagen) according to a 1:5 scale-down of the manufacturer’s instructions. Cytosolic proteins and proteins enriched in the plasma membrane, as well as in all organelles (including mitochondria) were sequentially isolated in the F1 and F2 fractions, respectively. Proteins from fractions F1 and F2 were separated by SDS-PAGE and analyzed by Immunoblotting. The following proteins were detected as markers specific for each fraction: HSP90 for the cytosolic fraction and Bcl-2 for the mitochondria-containing fraction.

For the isolation of the nuclear and cytoplasmic fractions to analyze PIN-1 localization we followed a different protocol. First, the cytoplasmic protein extract was obtained upon cell lysis with the following buffer, supplemented with proteases inhibitors: 10 mM Hepes pH 7.5, 40 mM KCl, 3 mM MgCl_2_, 5% glycerol, and 0.2% NP40. Then, nuclear extracts were obtained upon addition of the following buffer (supplemented with proteases inhibitors) to the resulting pellet: 20 mM Hepes pH 7.5, 420 mM NaCl, 1.5 mM MgCl_2_, 25% glycerol, 0.2 mM EDTA, followed by three cycles of freezing and thawing to disrupt nuclear membranes.

### In vivo experiments

CD/1 female athymic nude mice were purchased from The Jackson Laboratory and housed in groups of six or less in isolated ventilated cages; food and water were provided ad libitum. All animal procedures were performed according to protocol approved by the Istituto Superiore di Sanità Animal Care Committee.

2*10^5^ NIH-H460 cells were subcutaneously injected into the flank of 6–8 weeks old mice. After one week, when the tumors reached an average volume of 50 mm^3^, the tumor bearing nude mice were treated with PBS or LNA-663 at 15 mg/kg, by peri-tumoral injection two times per week for 2 weeks. Tumor diameters were measured at regular intervals as described above. Tumor size was assessed every 2 days by caliper measurement. Tumor volume was calculated as follows: *D*×*d*^2^×*π*/6, where *D* and *d* are the longer and the shorter diameters, respectively. PBS group *n* = 5; LNA-663 group *n* = 8.

## Discussion

MiRs contribute to cancer onset and progression in most human malignancies^42^. Some miRs can act as both oncogenes and oncosuppressors in a tissue-dependent manner as a consequence of differential gene expression pattern determining specific miR-target interactions. The dual role of miR-663 has been already documented in recent literature reporting oncosuppressive activity in glioblastoma^43,44^, pancreatic cancer^45^ and thyroid carcinoma^46^, and supporting the oncogenic function of this miR in prostate cancer^47^ and nasopharyngeal carcinoma^21^. In lung cancer it was reported that miR-663 is highly expressed in tumors compared to matched normal tissues and a pro-proliferative role of the miR was suggested^48^.

In this study, we identified miR-663 as potential oncogene in NSCLC cells. We performed an unbiased screening by monitoring miRs loss-of-function effect on cancer cell viability and selected miR-663 among the top hits. MiR-663 depletion resulted detrimental for lung cancer cells, supporting a pro-tumoral activity in this cellular context. This hypothesis was further verified in four NSCLC cell lines and in patient-derived cells, harboring different histological origins and mutational status, corroborating our previous findings. Furthermore, we performed in vivo experiments to establish the therapeutic potential of miR-663 ablation. We showed that LNA-663 treatment significantly reduces the growth of tumor xenografts in immunodeficient mice, providing encouraging bases for further development of a new therapeutic approach entailing miR-663 targeting. The analysis of miR-663 expression on patient-derived specimens did not reveal a higher expression in tumor versus normal samples and no correlation with patient’s prognosis was found when interrogating TCGA datasets. Further, overexpression of miR-663 by a miR-mimic did not increase lung cancer cells proliferation or resistance to chemotherapy (data not shown). Based on these observations, we conclude that miR-663 does not behave as a driver gene in lung cancer onset and/or progression, while it plays a key role in the control of apoptosis and is therefore necessary for lung cancer homeostasis.

Deepening our analysis, we unraveled the mechanisms of action of miR-663. We confirmed p21 as target of this microRNA, showing that it contributes to the control of cell fate by miR-663 in NSCLC. Further, we report a rapid induction of cell death following miR-663 neutralization that is consistent with direct activation of MOMP effectors. Interestingly, we found that miR-663 controls the escape from apoptotic impulses by negatively regulating the expression of two novel target genes: PUMA and BTG2. We observed mitochondrial cell death induction upon miR-663 depletion that was partially dependent on the function of the BH3-only protein PUMA. Noteworthy, the upregulation of PUMA is p53-independent as it does not occur at transcriptional level. Consistently, PUMA accumulation occurred also in p53-NULL cell lines such as Calu-1 and H1299 cells thus explaining the effect of LNA-663 in this background and and in p53-mutant patient-derived cells.

In a p53 wt background BTG2, the second target identified, cooperates with and empowers the action of PUMA on MOMP induction. BTG2 is an early response gene whose expression is induced upon several stress stimuli and regulates cell differentiation, proliferation, DNA damage repair, and apoptosis^28^. We recapitulated the mechanism of apoptosis induction exerted by BTG2 through the interaction with the cis–trans isomerase PIN-1, involving p53 stabilization, and mitochondrial localization. Moreover, we underlined the contribution of miR-663 upstream of these events, showing how miR-663-dependent regulation of BTG2 controls p53 mitochondrial translocation and by that programmed cell death. In NSCLC-bearing mutant p53, we found that BTG2 is not a relevant target of miR-663. By silencing BTG2 in miR-663-depleted cells, we were not able to rescue the induction of apoptosis, suggesting that PUMA and possibly other targets of miR-663 mediate the apoptotic cascade in the absence of p53.

 Our data demonstrate that miR-663 has a determinant role in apoptosis escape of NSCLC cells, thus sustaining cancer progression. One of the hallmarks of cancer cells is indeed the ability to escape control strategies meant to avoid aberrancies and uncontrolled proliferation, such as programmed cell death. Transformed cells are subjected to a number of apoptotic stimuli and very often develop alternative mechanisms to sustain cancer progression including the inhibition of MOMP, which is a crucial step of the intrinsic pathway of apoptosis^11,49^. BH3-only proteins are pro-apoptotic factors that antagonize the pro-survival members of the Bcl-2 protein family, which in turn exert an inhibitory action on MOMP. Here we showed how miR-663 controls MOMP induction by directly targeting the BH3-only protein PUMA and indirectly triggering a non-canonical function of p53, which behaves as a BH3-only protein itself upon mitochondrial translocation. Pharmacological agents that induce or facilitate MOMP represent attractive strategies for cancer therapy. Notably, BH3-only proteins are key effectors in the anti-cancer response to several therapeutic approaches, such as tyrosine kinase inhibition, HDAC inhibition, taxanes, glucocorticoids, and DNA-damaging drugs. Further, small molecules that mimic BH3-only proteins (BH3-mimetics) are currently under preclinical and clinical development^50,51^. In particular, ABT-263 (navitoclax), inhibitor of Bcl-2, Bcl-XL, and Bcl-W, and the less toxic BH3-mimetic targeting only BCL-2, ABT-199 (venetoclax), hold great promise in combinatorial treatment of several cancer types^52,53^. Our data support the development of new approaches to modulate BH3-only effectors by controlling the expression of miR-663 in NSCLC.

## Electronic supplementary material

Fig. S1

Fig. S2

Fig. S3

Fig. S4

Fig. S5

Fig. S6

Table S1

Table S2

Table S3

Supplementary Figure Legends
