# Supplementary figures and images for: miR-663 sustains NSCLC by inhibiting mitochondrial outer membrane permeabilization (MOMP) through PUMA/BBC3 and BTG2
Source: Cell Death Dis. 2018 Jan 19;9(2):49. doi: 10.1038/s41419-017-0080-x (PMC5833438; doi:10.1038/s41419-017-0080-x)

## Slide 1
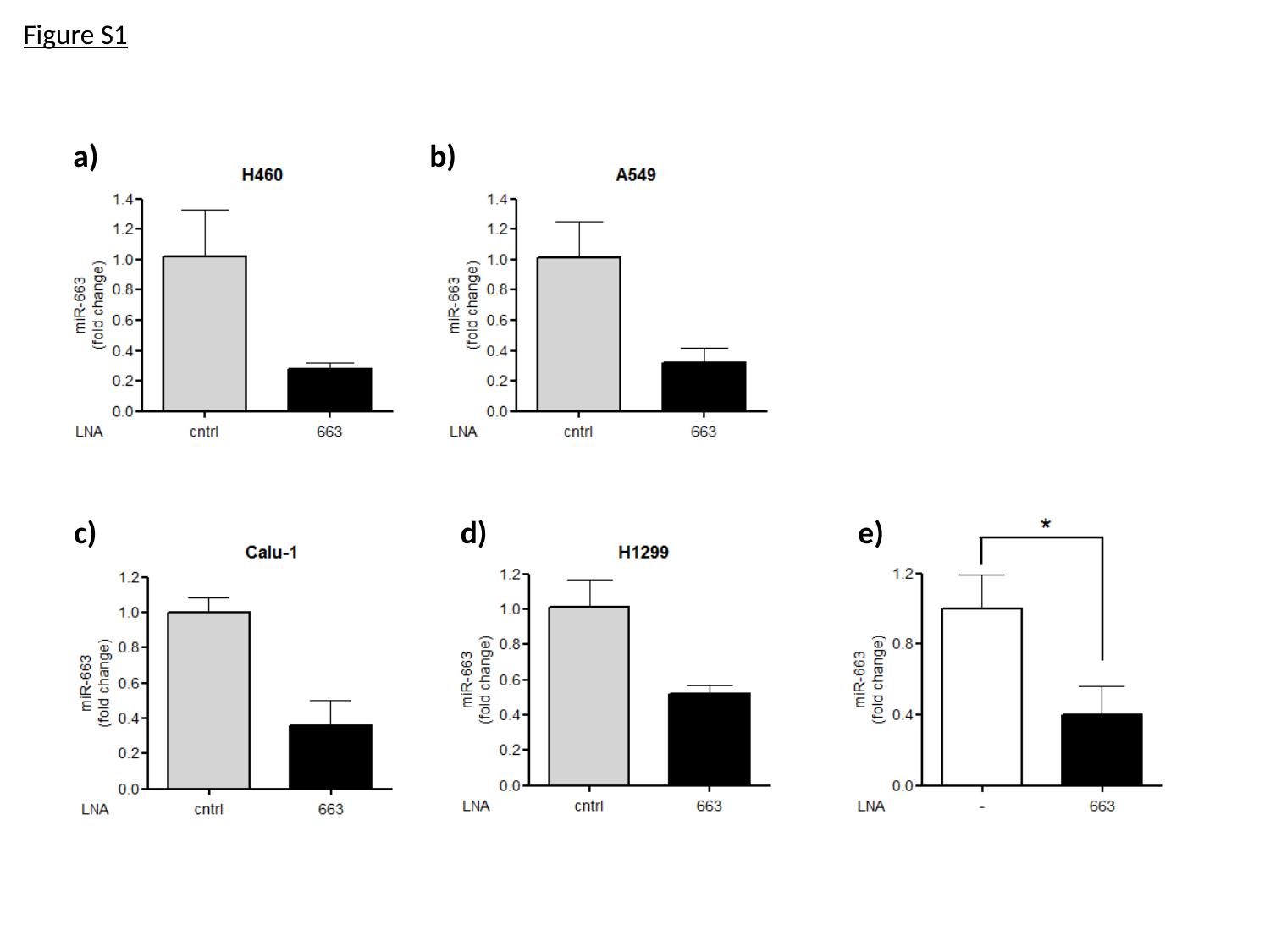

Figure S1
a)
b)
e)
c)
d)

Supplement: Supplementary file 1 — Fig. S1 [file 41419_2017_80_MOESM1_ESM.pptx]

## Slide 1
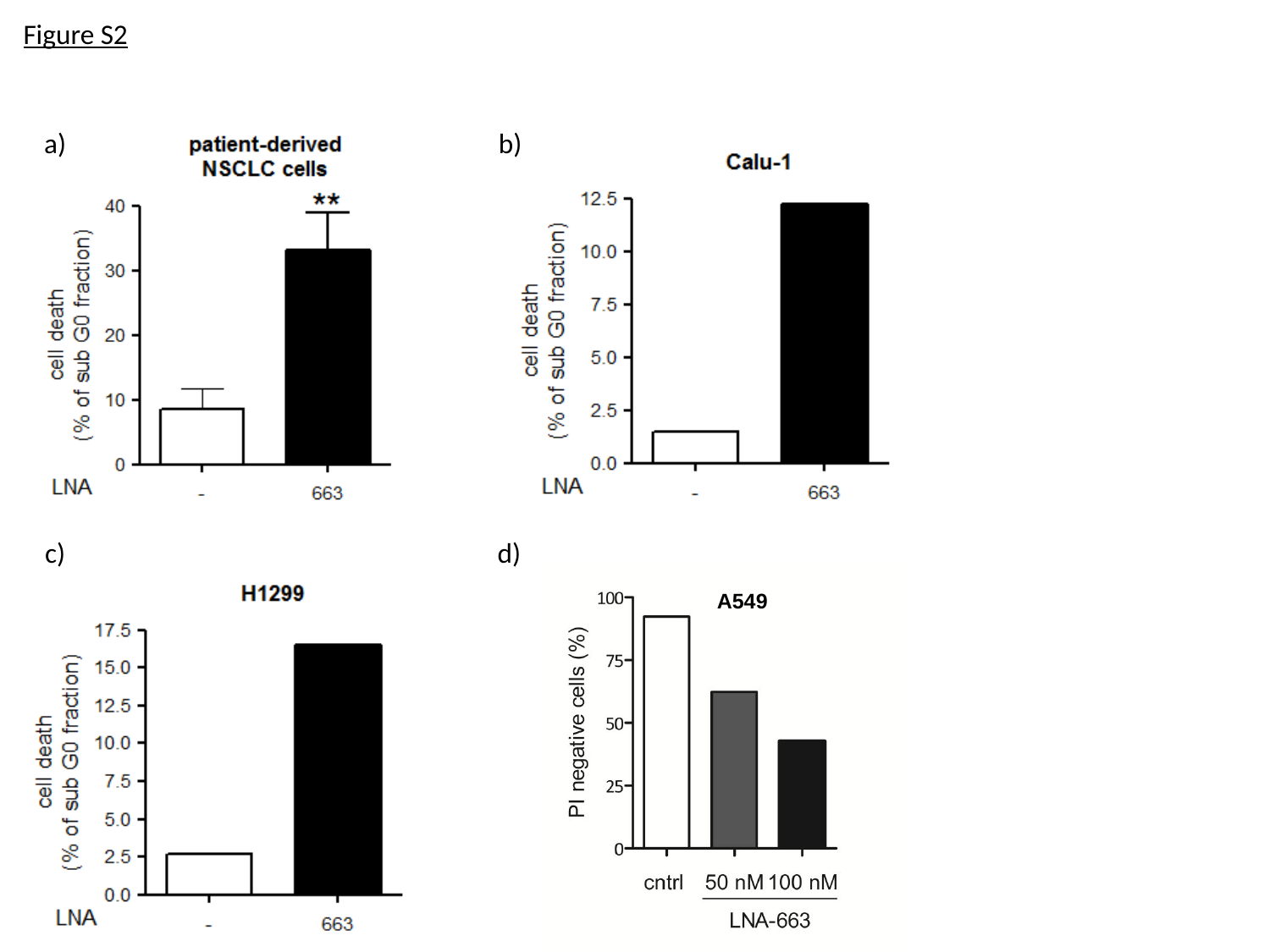

Figure S2
a)
b)
d)
c)
A549

Supplement: Supplementary file 2 — Fig. S2 [file 41419_2017_80_MOESM2_ESM.pptx]

## Slide 1
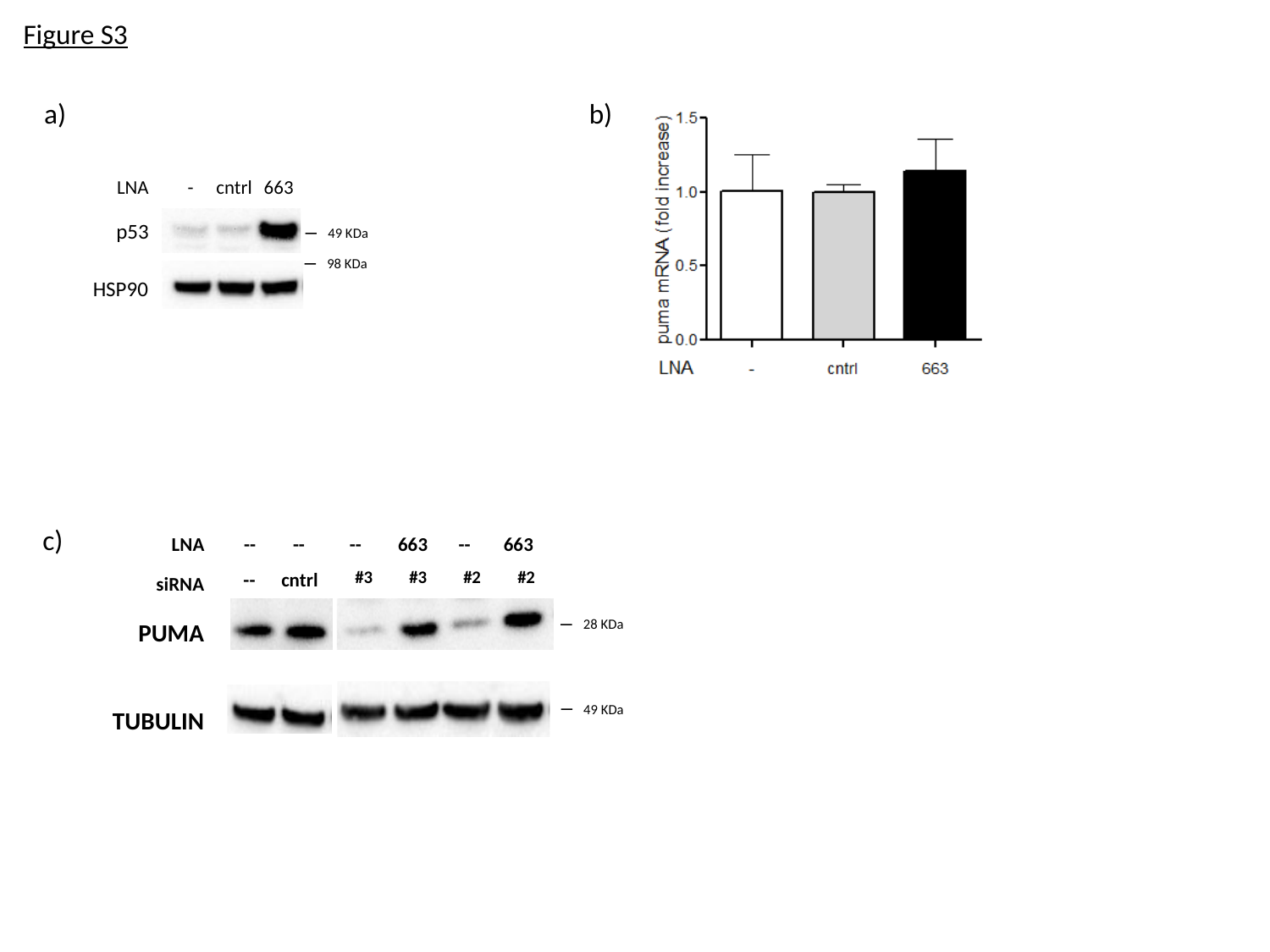

Figure S3
a)
b)
663
LNA
-
cntrl
p53
HSP90
49 KDa
98 KDa
c)
LNA
--
--
--
663
--
663
#3
#3
#2
#2
cntrl
--
siRNA
PUMA
TUBULIN
28 KDa
49 KDa

Supplement: Supplementary file 3 — Fig. S3 [file 41419_2017_80_MOESM3_ESM.pptx]

## Slide 1
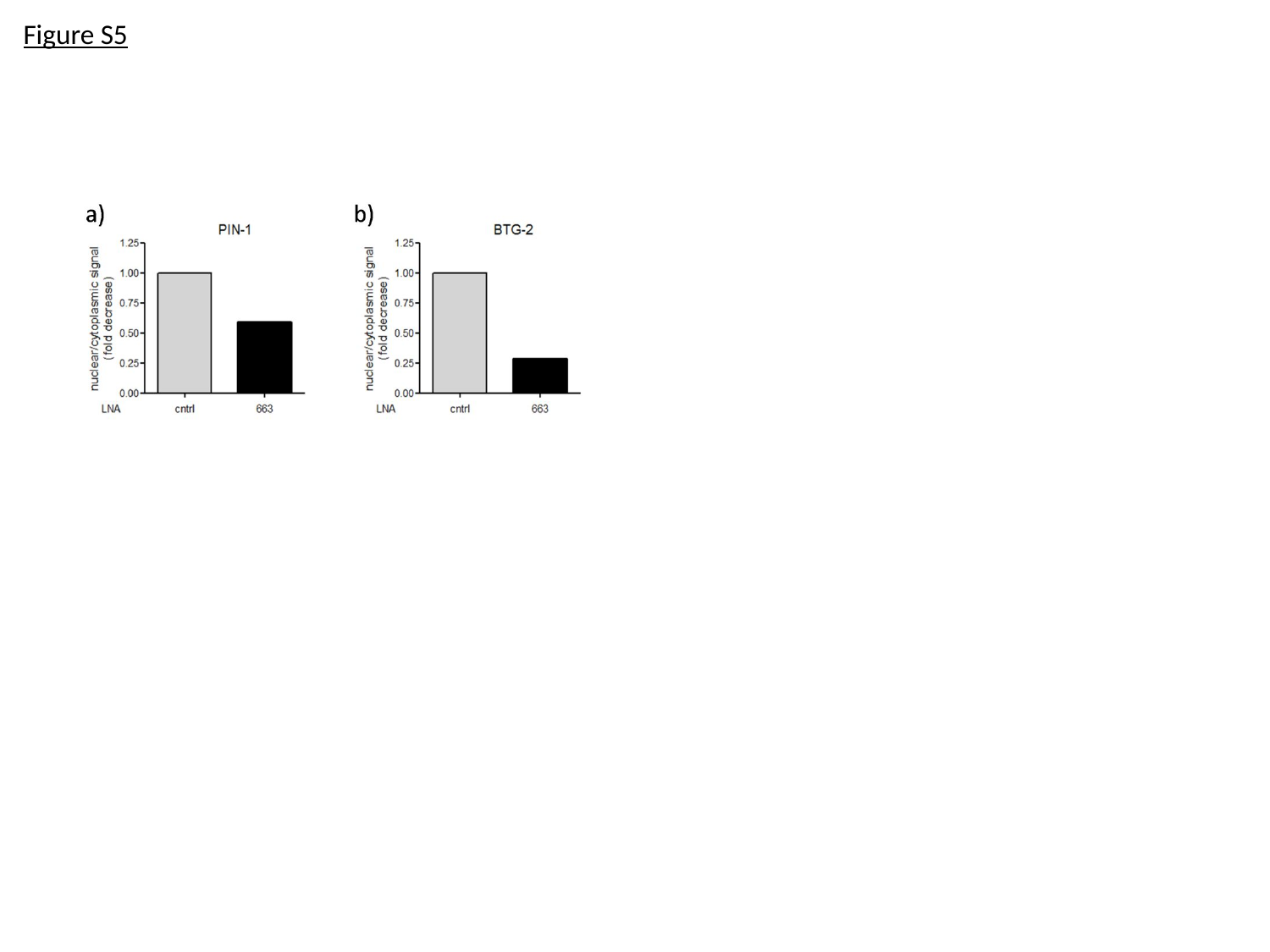

Figure S5

Supplement: Supplementary file 5 — Fig. S5 [file 41419_2017_80_MOESM5_ESM.pptx]

## Slide 1
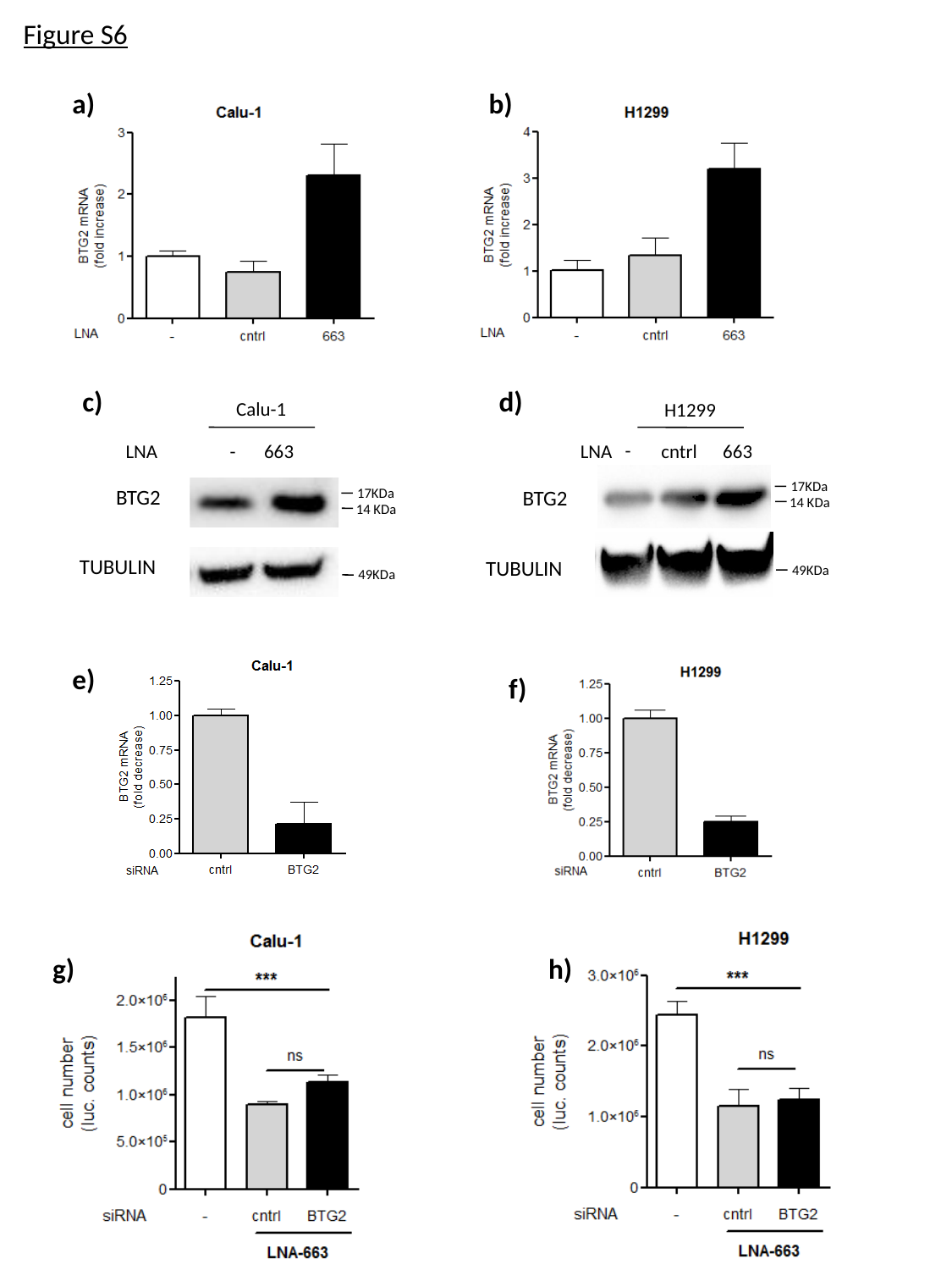

Figure S6
a)
b)
c)
Calu-1
-
 663
LNA
BTG2
17KDa
14 KDa
TUBULIN
49KDa
d)
H1299
-
 cntrl
 663
17KDa
14 KDa
49KDa
LNA
BTG2
TUBULIN
e)
f)
g)
h)

Supplement: Supplementary file 6 — Fig. S6 [file 41419_2017_80_MOESM6_ESM.pptx]
