# Supplementary material for: miR-663 sustains NSCLC by inhibiting mitochondrial outer membrane permeabilization (MOMP) through PUMA/BBC3 and BTG2
Source: Cell Death Dis. 2018 Jan 19;9(2):49. doi: 10.1038/s41419-017-0080-x (PMC5833438; doi:10.1038/s41419-017-0080-x)
Supplement: Supplementary file 4 — Fig. S4 [file 41419_2017_80_MOESM4_ESM.pptx]

## Slide 1
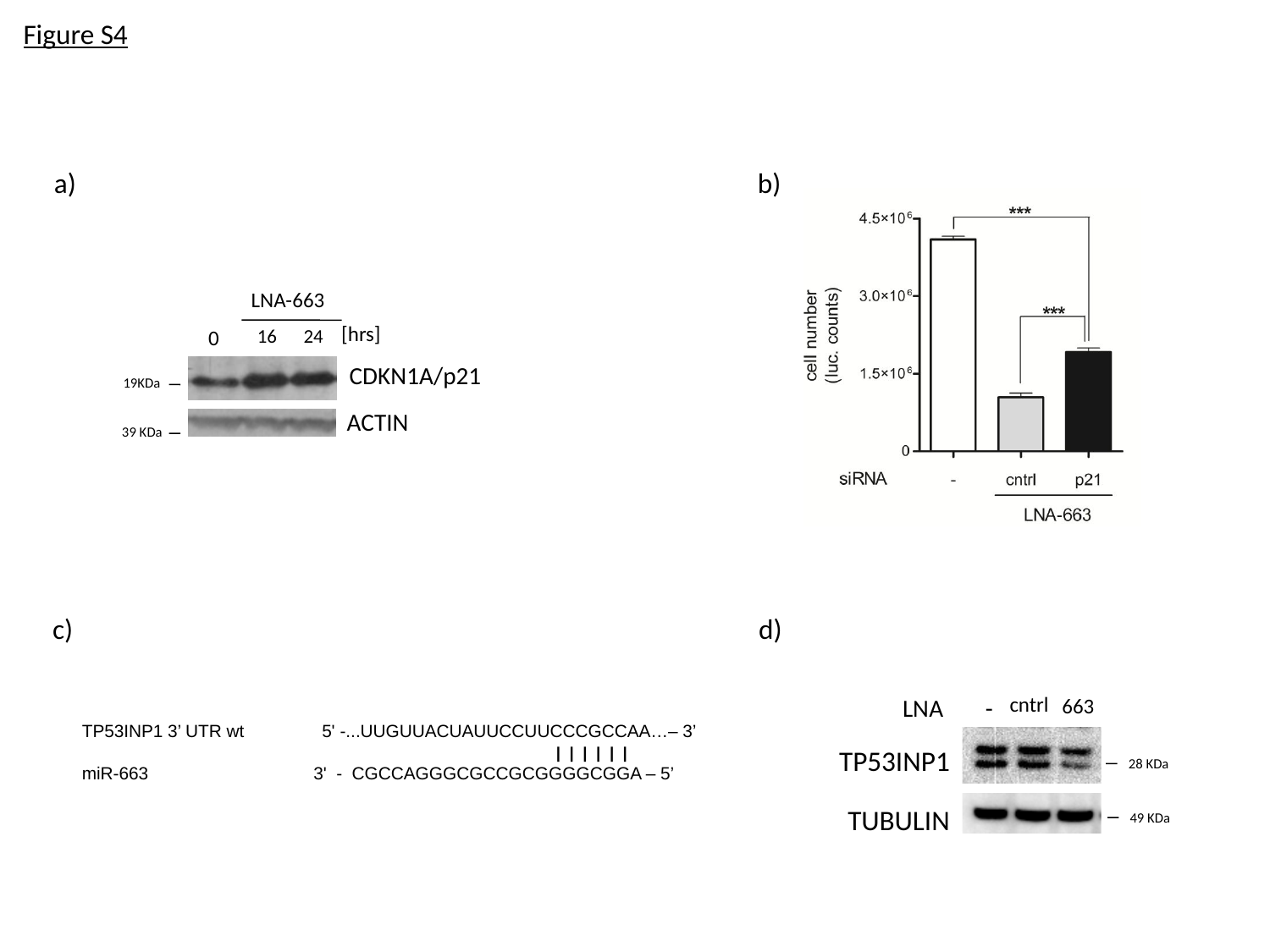

Figure S4
a)
b)
LNA-663
[hrs]
 16 24
0
CDKN1A/p21
ACTIN
19KDa
39 KDa
c)
d)
 cntrl
663
-
LNA
TP53INP1
TUBULIN
28 KDa
49 KDa
TP53INP1 3’ UTR wt 5' -...UUGUUACUAUUCCUUCCCGCCAA…– 3’
             	 | | | | | | 
miR-663 3'  -  CGCCAGGGCGCCGCGGGGCGGA – 5’
