# Supplementary material for: miR-663 sustains NSCLC by inhibiting mitochondrial outer membrane permeabilization (MOMP) through PUMA/BBC3 and BTG2
Source: Cell Death Dis. 2018 Jan 19;9(2):49. doi: 10.1038/s41419-017-0080-x (PMC5833438; doi:10.1038/s41419-017-0080-x)
Supplement: Supplementary file 10 — Supplementary Figure Legends [file 41419_2017_80_MOESM10_ESM.docx]

miR-663 sustains NSCLC by inhibiting

mitochondrial outer membrane

permeabilization (MOMP) through PUMA/

BBC3 and BTG2

*Figure S1*- *q-RT-PCR analysis of miR-663 expression upon LNA-663 treatment* on NIH-H460 cells **(a)** A549 **(b)**, Calu-1 **(c)** and H1299 **(d)**. Mean **±** s.d. One representative experiment is shown. **e)** miR-663 levels measured in LNA-treated mice (n=4). Mean **±** s.e.m. *p<0.05.

*Figure S2*- *LNA-663 treatment induces apoptosis in non small cell lung cancer cells –* **a-b-c)** FACS analyses of apoptotic cells - shown as sub G0 fraction - upon LNA-663 transfection in patient-derived NSCLC cells, Calu-1 and H1299 cells. **d)** FACS analysis of PI-stained A549 cells transfected with the indicated concentrations of LNA-663; **a-** mean of three independent experiments. Mean **±** s.d., **p=0.001445. **b-c-d** one representative experiment is shown.

*Figure S3*- **a)** Immunoblot showing p53 stabilization upon miR-663 neutralization in NIH-H460 cells. HSP90 was used as protein loading control. **b)** PUMA mRNA levels in NIH-H460 cells untreated and transfected with the indicated LNAs, were analyzed by real time PCR - GAPDH was used for PCR normalization - Mean **±** s.d.; **c)** Western blotting showing PUMA downmodulation in NIH-H460 cells treated with a control or two different anti PUMA siRNA (#2 and #3 Qiagen). Double transfection of anti PUMA siRNA together with LNA-663 restores PUMA levels of control cells, counteracting LNA-mediated induction. TUBULIN was used as protein loading control.

*Figure S4*- *p21 is regulated by miR-663 in NIH-H460 cells and contributes to its pro-tumoral role* **a)** Western blotting showing CDKN1A/p21 levels in NIH-H460 cells depleted for miR-663. **b)** Downmodulation of CDKN1A/p21 by RNA interference can rescue LNA-663 effect on NIH-H460 cells as shown by Cell Titer Glo assay. Mean **±** s.d., ***p<0.001.

TP*53INP1 is not a direct target of miR-663 -* **c)** Predicted TP53INP1 3’ UTR binding site for miR-663. The alignment of the seed region of miR-663 with TP53INP1 3’ UTR is shown. **d)** Western blotting showing TP53INP1 protein levels in NIH-H460 cells treated LNA-663. TUBULIN was used as protein loading control.

*Figure S5- quantitative analyses on IF images*. The ratio between nuclear and cytoplasmic signal as measured with Image-J is shown for PIN-1 **(a)** and BTG2 **(b)**.

*Figure S6- BTG2 role in p53-NULL cells.* **a-b).** BTG2 levels upon LNA-663 treatment were evaluated in Calu-1 and H1299 cells, by qPCR **(a-b)** and western blot **(c-d). e-f)** BTG2 downmodulation upon siRNA treatment is shown by qPCR. GAPDH was used for PCR normalization - Mean **±** s.d. **g-h)** Downmodulation of BTG2 by RNA interference does not rescue LNA-663 apoptotic effect in Calu-1 and H1299 cells as shown by Cell Titer Glo assay - Mean **±** s.d.***p<0.001, ns not significant.

*Table S1, related to Figure 1*. Histological origin and mutational status of lung cancer cells used in this study. Provided as an Excel file.

*Table S2, related to Figure 4.*Top 568 genes that resulted upregulated >1.5 fold from gene array analyses upon miR-663 knock down. Putative target genes predicted by TargetScan are indicated. Provided as an Excel file.

*Table S3, related to Figure 4*. Putative targets of miR-663 predicted by TargetScan. Provided as an Excel file.
